# Update on protease-activated receptor 2 in inflammatory and autoimmune dermatological diseases

**DOI:** 10.3389/fimmu.2024.1449126

**Published:** 2024-09-19

**Authors:** Kejia Xu, Lin Wang, Mao Lin, Gu He

**Affiliations:** ^1^ Department of Dermatology, West China Hospital, Sichuan University, Chengdu, China; ^2^ Laboratory of Dermatology, Clinical Institute of Inflammation and Immunology, Frontiers Science Center for Disease Related Molecular Network and State Key Laboratory of Biotherapy, West China Hospital, Sichuan University, Chengdu, China

**Keywords:** protease-activated receptor 2, signaling, immunity, inflammatory dermatological disease, comorbidity

## Abstract

Protease-activated receptor 2 (PAR2) is a cell-surface receptor expressed in various cell types, including keratinocytes, neurons, immune and inflammatory cells. Activation of PAR2, whether via its canonical or biased pathways, triggers a series of signaling cascades that mediate numerous functions. This review aims to highlight the emerging roles and interactions of PAR2 in different skin cells. It specifically summarizes the latest insights into the roles of PAR2 in skin conditions such as atopic dermatitis (AD), psoriasis, vitiligo and melasma. It also considers these roles from the perspective of the cutaneous microenvironment in relation to other inflammatory and autoimmune dermatological disorders. Additionally, the review explores PAR2’s involvement in associated comorbidities from both cutaneous and extracutaneous diseases. Therefore, PAR2 may serve as a key target for interactions among various cells within the local skin environment.

## 1 Introduction

Protease-activated receptor 2 (PAR2), first described in 1994 (1), is a versatile transmembrane receptor that senses and responds to active proteases in the cellular microenvironment. As a member of protease-activated receptor and a subfamily of G protein-coupled receptors (GPCRs), PAR2 shares several common structural features including an extracellular NH2-terminal domain, seven transmembrane helices, three extracellular loops, three intracellular loops, and an intracellular COOH terminus (2). Uniquely, PAR2 can be activated by various proteases from both endogenous sources (e.g.trypsin, mast cell-derived tryptase, kallikrein-related peptidases (KLKs), and coagulation proteases (3) such as thrombin, Factor Xa (FXa), FVIIa, FIXa), as well as membrane-type serine protease-1, human airway trypsin-like protease) and exogenous sources (e.g. bacteria, house dust mite (HDM), cockroaches, pollens, and molds), leading to a multitude of biological effects across various tissues and organ systems (4). To date, PAR2 has been widely expressed on epithelial cells, immune cells, neurons and so on, playing a critical role in homeostasis and in various disease processes, including asthma, lung injury, inflammatory bowel diseases, irritable bowel syndrome, neurogenic inflammation and cancer. In the skin and its microenvironment, functional PAR2 is primarily expressed in epidermal keratinocytes (KCs), and neighboring cells such as mast cells, eosinophils, neutrophils, dendritic cells, T cells and neurons, also exhibit PAR2 expression. Proteases like Der p3 and Der p9 from HDM (5), Per a7 from cockroaches allergens (6), KLK5, KLK14, trypsin are notable for proteolyzing PAR2, thereby mediating epidermal barrier homeostasis, innate and adaptive immunity, leukocyte recruitment, pigmentation, tumorigenesis and cutaneous paresthesia (7). Indeed, PAR2 appears to have a significant role in atopic dermatitis (AD), psoriasis, vitiligo, melasma, non-histaminergic pruritic skin disorders, syringoma and squamous cell carcinoma (4). Given the growing attention on inflammatory and autoimmune dermatological illnesses and their various cutaneous and extracutaneous comorbidities, PAR2 is considered a key target for facilitating cross-communication among different cells and tissues.

Previous reviews have examined the impacts of PAR2 on skin physiology and pathology (8, 9), however, to our knowledge, the detailed roles of PAR2 in inflammatory and autoimmune dermatological diseases have not yet been thoroughly investigated considering novel developments and emerging discoveries. Understanding the intricate connections, such as those involving resident skin cells and neurons expressing PAR2, could clarify the pathogenesis of diseases like AD, psoriasis, and vitiligo. Therefore, this review focuses on the latest updates on PAR2 and its potential effects in various cutaneous diseases from the perspective of the local cutaneous microenvironment. The dysregulation and abnormal expression of PAR2 in the cutaneous milieu may promote disease progression through cell-surface interactions, integration of extracellular signals, and induction of intracellular signaling pathways.

## 2 Activation, signaling and trafficking of PAR2

### 2.1 Protease-stimulated PAR2 activation

**Figure 1:**
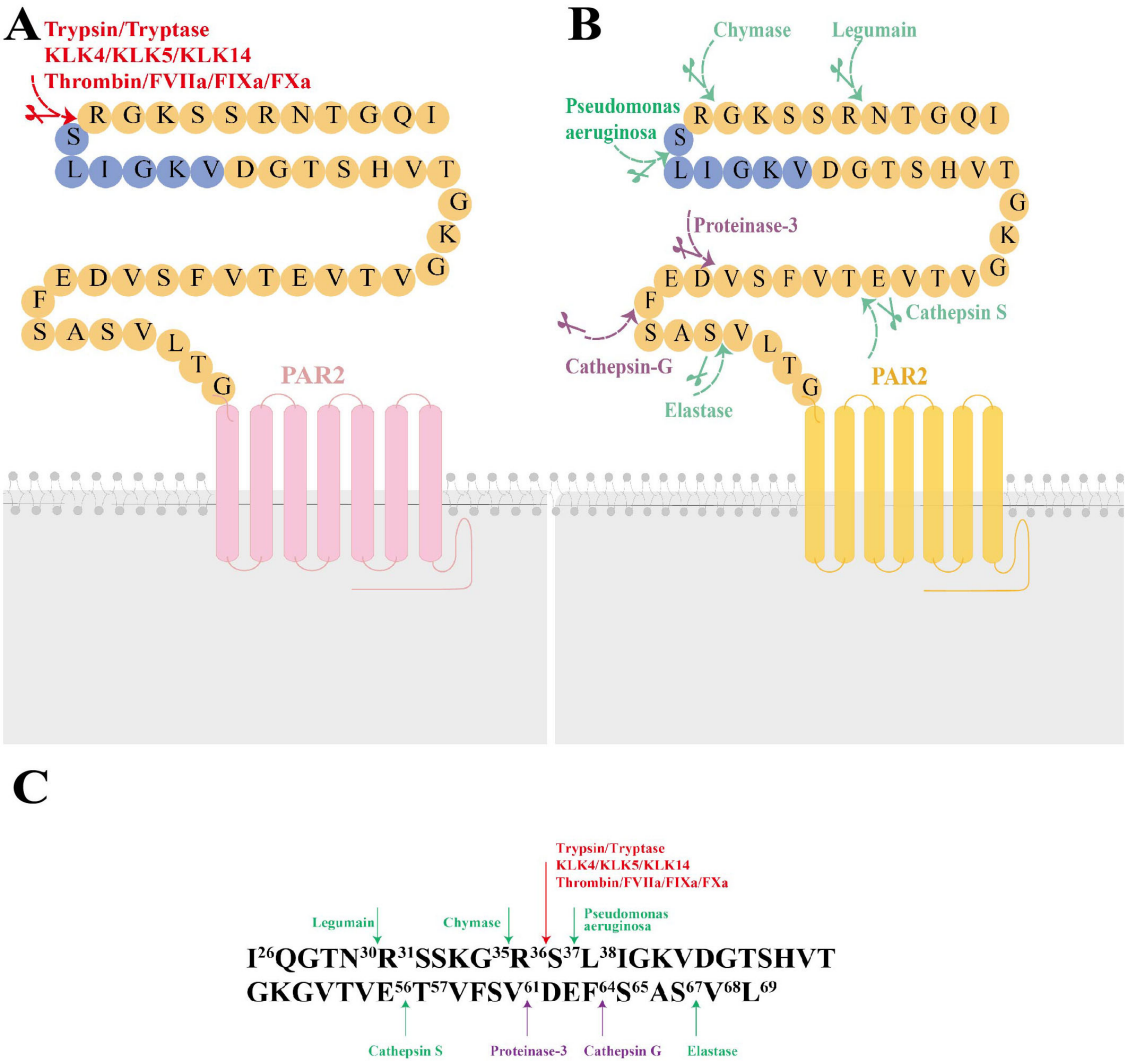
The molecular structure of Protease-Activated Receptor 2 (PAR2), its canonical and noncanonical activation mechanisms, and the responding proteases at the cleavage sites. **(A)** The molecular structure of PAR2 and its canonical activation by trypsin, tryptase, KLK4, KLK5, KLK14, FXa, FIXa, FVIIa, thrombin is illustrated. Canonical activation of PAR2 involves a proteolytic process that reveals the tethered ligand sequence at the Arg^36^/Ser^37^ site (highlighted in red). **(B)** Noncanonical activation of PAR2 includes cleavage at biased sites (highlighted in green) or generates disarming changes (highlighted in purple). **(C)** major activating proteases and cleavage sites of PAR2. KLK4, kallikrein-related peptidase 4; KLK5, kallikrein-related peptidase 5; KLK14, kallikrein-related peptidase 14; FXα, Factor Xα; FIXa, Factor IXa; FVIIa, Factor VIIa.

PAR2, a cell-surface receptor, primarily undergoes activation through proteolytic cleavage, which exposes a tethered ligand at specific extracellular N-terminal sites. The residues exposed from this cleavage bind to an extracellular docking domain, inducing a conformational change that triggers intracellular signaling. This proteolytic process, extensively studied and known as “canonical activation” was initially shown to involve trypsin cleaving mouse PAR2 at Arg^38^/Ser^39^ and human PAR2 at Arg^36^/Ser^37^, thereby exposing the tethered ligands SLIGRL and SLIGKV, respectively (10). Subsequent research have identified other serine proteases, including tryptase, KLK4, KLK5, KLK14, Thrombin (11), FVIIa, FIXa and FXa, which also hydrolyze PAR2 at canonical sites with slight variations (12) (**Figure 1A**). “Noncanonical activation” describes the selective activation of specific intracellular signaling pathways by distinct ligands that cleave at biased sites or cause a conformational change in the receptor sufficient for activation (**Figure 1B**). These cleavage sites are either proximal or distal to the canonical sites (13). For instance, cysteine proteases Legumain and Cathepsin S cleave PAR2 at Asn^30^/Arg^31^ (proximity) and Glu^56^/Thr^57^ (distality), thus exposing distinct tethered ligands RSSKGR and TVFSVDEFSA, respectively (14, 15). Elastase similarly activates PAR2 by cleaving the receptor at Ser^67^/Val^68^ in the extracellular N-terminal region (15). Additionally, the serine protease chymase disrupts intestinal epithelial barrier via a biased mechanism by cleaving PAR2 at Gly^35^/Arg^36^ (16). In a previous study by Dulon et al (17), Pseudomonas aeruginosa cleaved PAR2 at Ser^37^/Leu^38^, thereby revealing LIGKV and disrupting the canonical tethered ligand. Similarly, Rayees et al. discovered later that pseudomonas aeruginosa interacted with alveolar macrophages, activating PAR2 and thereby affecting the macrophages’ ability to phagocytize the bacteria (18) (**Figure 1C**). Synthetic peptides, known as activating peptides, can activate PAR2 directly without proteolysis, mimicking the activation pathways mentioned above. They have been designed to mimic the effects of proteases, facilitate the investigation of PAR2 functions and develop selective ligands (agonists or antagonists). Given the varying effects of different ligands on PAR2, biased signaling is likely to be preferred for developing targeted drugs (19). Therefore, multiple natural and corresponding synthetic ligands can activate PAR2 at biased sites.

### 2.2 Signaling

**Figure 2:**
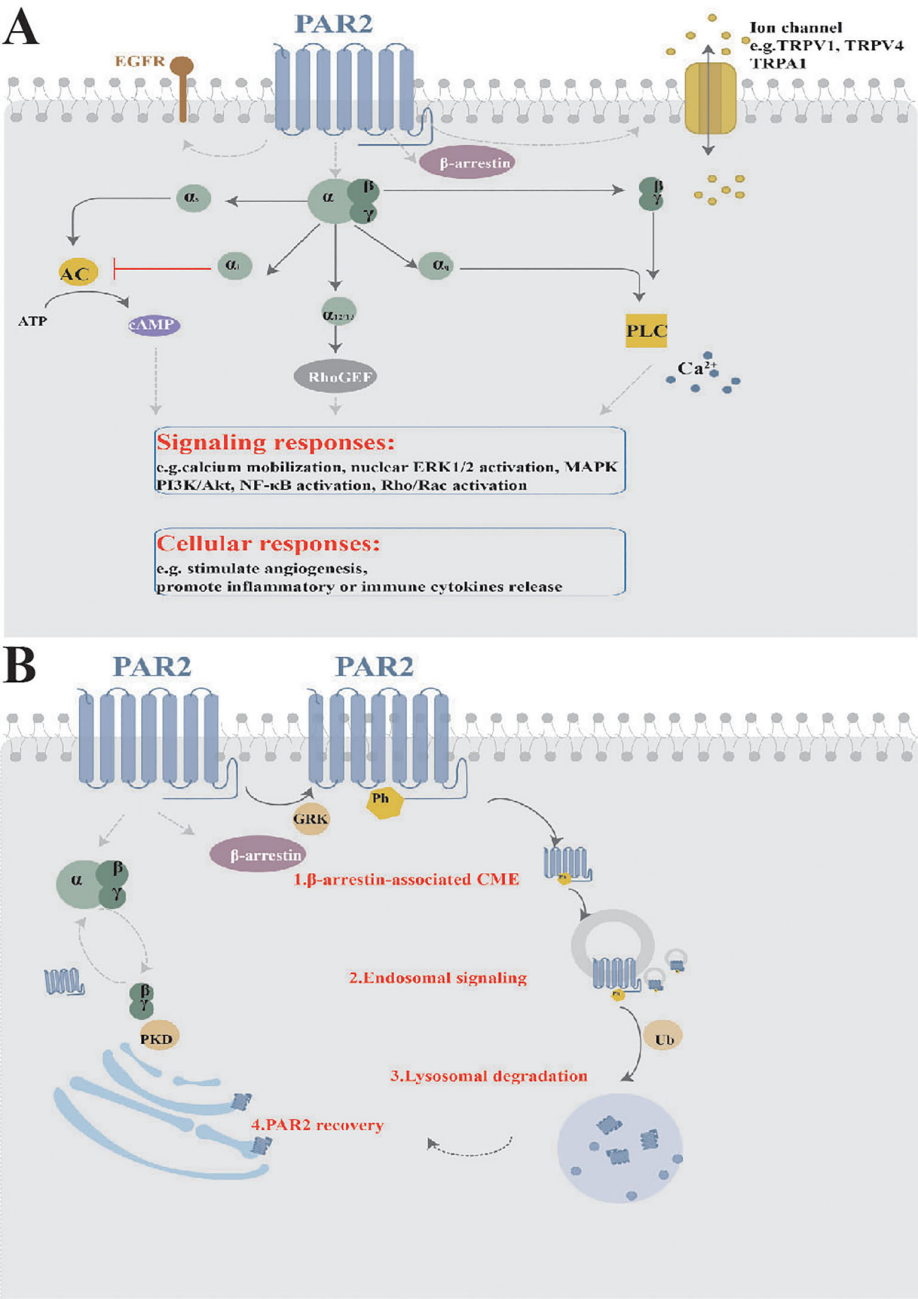
Signaling and Trafficking of PAR2. **(A)** Once activated, PAR2 initiates multiple downstream signaling cascades, including various intracellular signaling pathways, interactions with other receptor tyrosine kinases, and ion channel activation or releases, resulting in diverse cellular responses. **(B)** PAR2 trafficking involves processes such as endocytosis, degradation, and receptor recovery. MAPK, mitogen-activated protein kinases; EGFR, epidermal growth factor receptor; TRPV1, transient receptor potential vanilloid 1; TRPV4, transient receptor potential vanilloid 4; TRPA1, transient receptor potential ankyrin 1; NF-κB, nuclear factor kappa-B; PKD, Protein kinase D; GRK, G protein-coupled receptor (GPCR)-regulated kinase; CME, clathrin-mediated endocytosis; Ph, phosphorylation; Ub, ubiquitination.

Upon activation, PAR2 initiates multiple signaling cascades essential for maintaining homeostasis in physiological and pathological processes (**Figure 2A**). These cascades regulate cytokine production, stimulate angiogenesis, and promote inflammatory and immune responses (20). The downstream signaling pathways are complex and varied based on factors including specific hydrolytic positions, types, kinetics, potency, and post-translational modifications of PAR2. For example, at a concentration of 1 nM, tryptase efficiently cleaved the PAR2 at Arg^36^/Ser^37^. However, at 100 nM, while tryptase still cleaved at this site, it could target additional sites, potentially inhibiting the efficiency of PAR2 activation (21). The glycosylation of PAR2 may impact its susceptibility to tryptase activation. Key phosphorylation sites (Ser^383-385^, Ser^387^-Thr^392^) on the C-tail and the palmitoylation site (Cys^361^) on helix-8 of PAR2 also influence subsequent intracellular signaling cascades (22). Proteolytic disarming of PAR2 at biased sites, achieved by permanently removing canonical proteolysis and destroying the tethered ligand sequence, further enhances signaling complexity due to alteration in the typical signaling response (17).

Activated PAR2 engages multiple G protein-dependent and β-arrestin-associated pathways. The Gα subunit and Gβγ dimers separate from heterotrimeric G proteins. Different Gα subtypes include Gα_s_-regulated or Gαi-mediated AMP, Gα_12/13_-dependent Rho-Kinase activity, and Gα_q_-mediated Ca^2+^ release from the endoplasmic reticulum (23). In particular, once PAR2 is activated, rapid and transient Gα_q_-regulated Ca^2+^ release occurs, leading to the phosphorylation of mitogen-activated protein kinases, such as ERK1/2, and PI3K/Akt signaling (24). Mutated versions of PAR2 can stimulate intracellular MAPK pathways without Gα_q_ activation by cleaving other tethered ligands and corresponding soluble agonist peptides (25). The β-arrestin-associated signaling will be narrated in the next paragraph. Activation of PAR2 by trypsin results in Ca^2+^ mobilization, cAMP formation, and Rho-Kinase activity regulation by initiating Gα_q_, Gα_s_, Gα_12/13_, and recruiting β-arrestin, therefore rendering PAR2 internalization and degradation. In contrast, Cathepsin S only stimulates Gα_s_-mediated AMP formation, without Gα_q_-dependent Ca^2+^ signaling or β-arrestin recruitment (26). Cathepsin S also interacts with PAR2 to trigger additional Ca^2+^-dependent release through transient receptor potential (TRP) ion channels, bypassing the Gαq-mediated Ca^2+^ pathway in Xenopus laevis oocytes and mouse Dorsal Root Ganglion (DRG) neurons (15). Here, the Ca^2+^ released from intracellular stores, particularly from the Golgi apparatus upon PAR2 activation via trypsin, cathepsin-S, and neutrophil elastase, travels to the plasma membrane, where it plays a vital role in maintaining cellular signaling and ensuring the cell’s responsiveness to extracellular cues. Elastase, cathepsin-G, and proteinase-3 fail to activate Gαq-coupled PAR2 calcium signaling, while Legumain and its activating peptide lack β-arrestin recruitment but still participate in other cellular signaling mechanisms (14). Therefore, the activation modes and distribution of PAR2 in different tissues and cell types further influence intracellular signaling pathways.

Furthermore, downstream signaling cascades of PAR2 include the interactions with other receptor tyrosine kinases (e.g. epidermal growth factor receptor, platelet-derived growth factor receptors, vascular endothelial growth factor), TRP ion channels (e.g. transient receptor potential vanilloid 1 (TRPV1), TRPV4, and transient receptor potential ankyrin 1 (TRPA1)), and alternative gene expression (e.g. NF-κB, Toll-like receptor 4 (TLR4)) (27–30). Numerous studies have shown that active PAR2 sensitizes TRPV1, TRPV4, and TRPA1 channels, which are responsible for neuro-inflammation and pain (31, 32). The interaction between PAR2 activation and TRP ion channels results in sustained Ca^2+^ influx from both the extracellular region and endoplasmic reticulum, elevating intracellular Ca^2+^ levels and exacerbating physiological and pathological effects. Moreover, microarray analysis has identified hundreds of genes downstream of PAR2 signaling related to cellular metabolism, cell cycle, MAPK pathway, inflammatory cytokines, and anti-complement function (33).

### 2.3 Trafficking

Upon interaction with pericellular proteases, PAR2 becomes rapidly desensitized and irreversibly hydrolyzed, rendering it unresponsive to similar proteases or their activating peptides. For example, treating neurons with PAR2 agonists results in desensitization of the receptor, abolishing its interaction with trypsin or tryptase (34). Additionally, PAR2 is predominantly phosphorylated at multiple COOH-terminal domains (e.g. Ser/Thr residues) by GPCR kinases, crucial for β-arrestin recruitment and receptor endocytosis (35). β-arrestin recruitment occurs within minutes of PAR2 activation (36). PAR2 then undergoes uncoupling and clathrin-mediated endocytosis (37), internalizing into early endosomes through Rab5a, causing sustained endosomal signaling (38). Finally, PAR2 is ubiquitinated and targeted to lysosomes for degradation (39). However, PAR2 can recover at the cell surface from the Golgi in Rab11-dependent, Gβγ and PKD dependent manner (40). The trafficking patterns are typical (**Figure 2B**), but biased activation proteases do not involve β-arrestin recruitment, indicating that PAR2 signaling transport is not fully elucidated.

In summary, the activation of PAR2 at different sites by endogenous and exogenous proteases results in various intracellular and extracellular signaling cascades. These processes enable the adjustment of cellular responses to microenvironmental variations. Interestingly, some reports have revealed that activating PAR2 also suppresses inflammation. Two studies clarified that PAR2 activated by thrombin inhibited calcium ion signaling thereby reducing TLR4-induced inflammatory signaling (11) and Pseudomonas aeruginosa bound to PAR2 enhanced the clearance of bacteria therefore preventing fatal outcomes in bacterial pneumonia separately (18). Both studies were particularly related to innate immunity. In addition, Dr. Ruf and his team developed PAR2 mutant mice, including PAR2-deficient (PAR2−/−) models, to study PAR2’s specific roles in breast cancer progression, angiogenesis, diet-induced obesity and related metabolic disorders. Their studies are crucial for investigating biased PAR2 signaling and PAR2-dependent β-arrestin pathways, providing insight into PAR2’s unique functions (41–43). From the above elaboration, We infer that PAR2 activation, whether through canonical or non-canonical pathways, can result in similar or opposing effects, depending on the activation mechanism, receptor cleavage sites, and downstream signaling. Thus, A deeper understanding these mechanisms could offer opportunities for developing targeted therapies, potentially improving treatment efficacy for diseases characterized by dysregulated PAR2 signaling.

## 3 Function of PAR2 in skin

**Figure 3:**
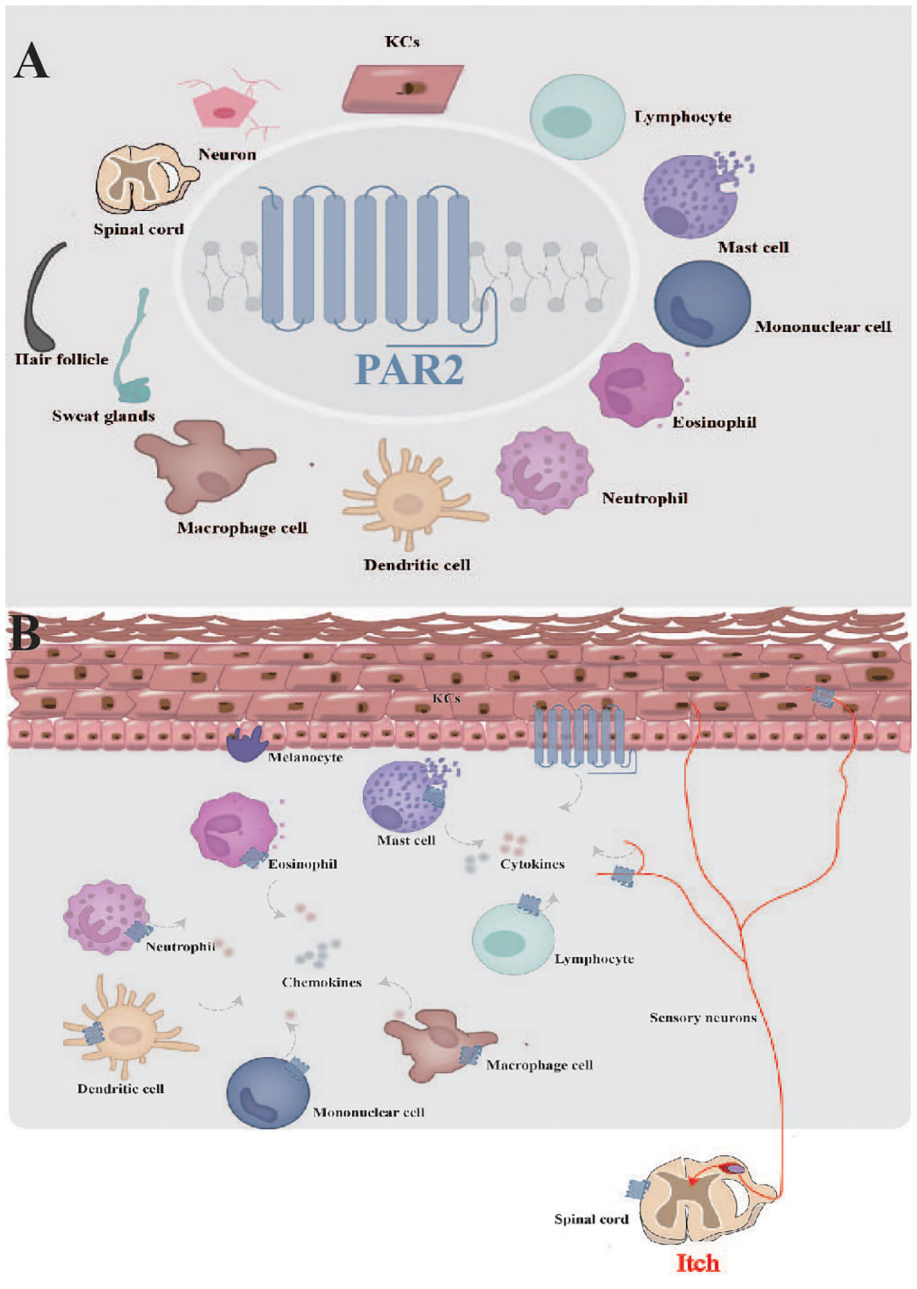
Function of PAR2 in skin. **(A)** PAR2 is widely expressed in the cutaneous microenvironment. **(B)** PAR2, expressed by various cells within cutaneous microenvironment, serves as a complex target for interactions among sensory neurons, resident skin cells, and transiently infiltrating cells. KCs, keratinocytes.

PAR2 expression has been detected in a diverse set of cell types within the cutaneous microenvironment, such as keratinocytes in the epidermis, and mast cells, eosinophils together with neurons in the dermis and subcutaneous tissue (**Figure 3A**). These cells interact and collectively influence skin inflammation, immune response and itching sensations. In the following sections, we will delve into the different types of cells expressing PAR2 and their respective functions.

### 3.1 PAR2 in keratinocytes

Keratinocytes make up approximately 95% of the epidermis and play a major role in maintaining the epidermal permeability barrier, mediating inflammation and immune responses, and regulating pigmentation. In human keratinocytes, PAR2 expression is significantly higher in the granular layer and is further enhanced in inflamed skin (10). Importantly, PAR2 localizes to lipid rafts in both human and murine keratinocytes (44). Studies in mice have shown that strong PAR2 expression in the epidermis during embryonic development, starting as early as embryonic day 17 (45). Here, the role of PAR2 in keratinocytes and the possible effects PAR2 on them are summarized:1. Cutaneous Barrier Function: PAR2 expressed in KCs regulates the epidermal barrier by initiating cytoskeletal rearrangements, modifying plasma membrane dynamics in response to barrier disruptions. Moreover, application of SLIGRL (an exogenous PAR2 agonist) protects and rapidly repairs the skin barrier (46). 2. Inflammation and Immune Responses: PAR2 could activate a wide variety of inflammatory cytokines and chemokines following the disruption of the epidermal permeability barrier. Moniaga et al. (47) found that when disrupted epidermal barrier occurred, activation of PAR2 led to the production of thymic stromal lymphopoietin (TSLP), a Th2-skewing skin inflammation and basophil accumulation were subsequently observed. These processes were suppressed by a PAR2 antagonist. Similarly, epidermal KLK5 (a serine protease for PAR2) directly activated PAR2, leading to a Th2 environment because of a series of cytokines and chemokines production (e.g. IL-1, TNF-α, GM-CSF, TSLP) (48). Hou et al. also revealed that both trypsin and SLIGKV could stimulate the chemokines like IL-8 secretion (49). 3. Pigmentation Regulation: In the absence of PAR2 expression in melanocytes, the effects on pigmentation are therefore attributed to melanosome transfer and paracrine melanogenesis, which are mediated by keratinocyte-melanocyte interactions. Darker skin exhibits higher levels of epidermal PAR2 compared to lighter skin due to more remarkable melanocore and melanosomes uptake by keratinocyte phagocytosis (50, 51). Kim et al. discovered that PAR2 also induced melanogenesis by stimulating stem cell factor from keratinocytes (52). 4. Cell Proliferation and Differentiation: Activated PAR2 inhibits keratinocyte growth with growth factor-supplemented or growth factor-free conditions (53). However, the involvement of PAR2 in keratinocyte differentiation is equivocal. Some research suggests that PAR2 activation promotes differentiation, while others indicate opposite outcomes. As the same study by Derian et al., both SLIGRL (a PAR2 selective agonist) and SFLLRN (a PAR2 stimulator) decreased differentiation by low expression of involucrin and transglutaminase type I (differentiation markers of keratinocytes) (53). Another study demonstrated that in primary human keratinocytes, decreased markers of differentiation were observed after PAR2 activation (54). Conversely, PAR2 promoted differentiation of keratinocytes when the epidermal barrier was compromised (55). The cause of the discrepancy remains unclear. Considering PAR2’s known ability to trigger intracellular calcium release, earlier researchers speculated that the lower differentiation mediated by PAR2 might be because the epidermis is a stratified epithelium, unlike other tissues (53). Recent findings from professor Piran’s team suggest that PAR2’s dual functions, depending on the activation site, could explain this contradiction (56). They propose that initial activation of PAR2 in the immune system exacerbates injury and inflammation, while if PAR2 is activated later within affected tissues, it promotes healing and regeneration. In their studies on liver regeneration models, they found that PAR2’s effects depend on the type of injury: it exacerbated immune-mediated damage but aided in regeneration following direct tissue injury (57). This dual role was confirmed in autoimmune diabetes and appeared to be consistent across various tissues (58). Although keratinocyte differentiation involves limited tissue regeneration, the conflicting roles of PAR2 in this process underscore its complex functions and suggest that PAR2 may have different effects within the same tissue type. Interestingly, we found that PAR2 promotes keratinocyte differentiation when the skin barrier is compromised. We speculate that broader inflammatory and immune responses, exacerbated by PAR2-mediated disruption of the skin barrier, may account for its varying effects on keratinocytes. Further research is needed to validate this.

In summary, PAR2 expressed in keratinocytes induces various and interactive functions: it negatively impacts the barrier function and cell proliferation, positively influences pro-inflammatory cytokine release and pigmentation, but has contradictory effects on cell differentiation.

### 3.2 PAR2 in immune and inflammatory cells

Activation of PAR2 expressed in keratinocytes, can significantly impact various immune and inflammatory cells (e.g. mast cells, eosinophils, lymphocytes, mononuclear cells, neutrophils, macrophages and dendritic cells), leading to complex immune and inflammatory responses. Interestingly, these cells also express PAR2, potentially amplifying and complicating these reactions. Mast cells (MCs), which contain tryptase, express PAR2 on the plasma membrane and intracellular granule membranes (59). Upon activation, it induces histamine or IL-8, thereby exacerbating inflammation and immune responses (60). In the latest research, the tryptase/PAR-2 axis has been identified as a critical component of the crosstalk between MCs and keratinocytes in skin inflammation (61). Analogously, the tryptase/PAR2 axis contributes to the hyperpigmentation of cutaneous lesions in mastocytosis without enhancing melanocyte activity (62). PAR2 is strongly expressed in human peripheral blood eosinophils, and tryptase from MCs could activate eosinophils to generate IL-6, IL-8 and leukotrienes. The release of IL-6 and IL-8 can be prevented by a PAR2 antagonist in a concentration-dependent manner (63). PAR2 also promotes neutrophils recruitment and upregulates IL-17 receptor signaling, along with promoting chemokines and cytokines (e.g. IL-23 and CXCL2) (64, 65). Similarly, PAR2 induces dendritic cells (DCs) maturation and may play a role in DCs trafficking to lymphnodes, thereby enhancing immune response (66). The influences of PAR2 on T lymphocytes are complicated. PAR2 is located on human CD4^+^ T cells and natural killer cells but not on CD8^+^ or γδT cells (67). However, activation of PAR2 in lymphocytes leads to the release of reactive oxygen species (ROS) (68). Thus, PAR2 is expressed by most immune cells in both the innate and adaptive immune systems, contributing to allergic inflammation and immunity. The co-expression and co-regulation of PAR2 among different cells may influence the intensity, duration, and the outcome of immune-inflammatory responses. Importantly, PAR2 is proposed as a potential target for the treatment of related diseases due to its significant role in modulating immune and inflammatory responses.

### 3.3 PAR2 in neurons

The dermis and subcutaneous tissue contain a complex network of nerves intertwined with various cell types responsible for sensory perception and the regulation of inflammatory cytokines. PAR2 is expressed by peripheral nerve endings, trigeminal ganglia, and primary spinal afferent neurons in the dorsal root ganglia, implicating it in neurogenic inflammation and sensation perception (69). Activation of PAR2 can induce edema and neutrophil infiltration by releasing calcitonin gene-related peptide and substance P (70). Additionally, PAR2 can sensitize TRPV1 by phosphorylation, amplifying intracellular processes (71). Gu et al. found that HDM allergens significantly enhanced TRPV1 in mouse pulmonary sensory neurons (72). Various studies suggest that PAR2 directly evokes pain, though evidence regarding its role in pruritus and thermal hyperalgesia is controversial. Initially, Vergnolle et al. discovered that PAR2 agonists induced both thermal and mechanical hyperalgesia (73). Nevertheless, Hassler et al. illustrated that in mice with PAR2 deleted in all sensory neurons, PAR2 expression in sensory neurons is merely responsible for pain-related behaviors, but not for thermal hyperalgesia or itch. The pain-relevant effects may be attributed to the mediation of the ERK signaling pathway activity (74). Furthermore, intradermal injection of PAR2 agonists can induce scratching behavior and activate neurons in the superficial dorsal horn of mice, indicating a role for PAR2 in the perception and signaling of itch at the neuronal level (75). These differences may be due to the peripheral and central innervation targets of PAR2-expressing neurons, as well as the sufficient proportion of these neurons to elicit different sensations. Furthermore, based on the study by Piran et al. (56–58), we infer that the debate over whether PAR2 triggers pruritus and thermal hyperalgesia, might hinge on if PAR2 is first activated within the immune system or not.

### 3.4 PAR2 in other cells

Besides, it has been found that skin appendages, such as hair follicles and myoepithelial cells of sweat glands, express PAR2 to varying extents (16). In addition, both trypsin and synthesized PAR2 agonists significantly enhanced the migration, adhesion, and proliferation of fibroblasts and macrophages, underscoring its crucial role in wound healing (76).

Together, the roles of PAR2 in the skin are complex and interrelated, given its varied distributions within the cutaneous microenvironment (**Figure 3B**). Elucidating the mechanisms involving PAR2 may provide important insights into the general understanding of this class of receptors in the skin.

## 4 PAR2 in inflammatory and autoimmune dermatological diseases

To date, numerous reports have demonstrated the dysregulation of PAR2 in inflammatory and autoimmune dermatological diseases, suggesting it as a potential marker. This discussion predominantly focuses on the impacts of PAR2 in atopic dermatitis, psoriasis, vitiligo, melasma, and other conditions such as rosacea, acne, and dermatomyositis, as summarized in **Table 1**. Therefore, potent PAR2 agonists and antagonists have emerged as enticing therapeutic agents although they are currently still in the experimental stage, primarily tested in genetically engineered mouse models.

**Table 1:** Summary of effects of PAR2 on inflammatory and autoimmune dermatological diseases.

Relevant diseases	Hypothesized effect		PAR2 perplexing effects		location	Potential mechanism	Role	Indication
	Promotes	Inhibits	aggravator-activated primarily in the immune system	alleviator-activated primarily in the affected tissue				
Atopic dermatitis(AD)	√				epidermal keratinocytes and peripheral nerves	promotion of Th2 cytokines (e.g. IL-4, IL-13 and TSLP) and GM-CSF	Th2 inflammatory response; induction of immunity; initiating neurogenic inflammation; disrupting epidermal barrier function;	(82–84)
Atopic dermatitis(AD)	√				epidermal keratinocytes	exaggerating neuro-epidermal communication	disrupting epidermal barrier function; initiating neurogenic inflammation	(85)
Atopic dermatitis(AD)	√				nerve fibers	increasing CGRP, substance P and releasing ion channels	neurogenic pruritus(histamine-independence)	(86)
Atopic dermatitis(AD)	√		√		epidermal keratinocytes and mast cells	promoting chemokines and cytokines	promoting barrier defects, allergic inflammation and pruritus	(87)
Psoriasis		√			epidermal keratinocytes	promoting cytokines (e.g. IL-8), accumulating epidermal inflammatory cells	inducting immunity and inflammation	(93)
Psoriasis	√		√		mast cells	promotion cytokines (e.g. IL-8)	inducing immunity and inflammation	(94)
Psoriasis	√				epidermal keratinocytes	promoting cytokines and chemokines	inducing immunity, inflammation and pruritus	(95)
Vitiligo		√			epidermal keratinocytes	antioxidant responses (e.g.Nrf2, MDA level)	transferring melanosomes	(100–102)
melasma	√				epidermal keratinocytes	formation of telangiectatic erythema	inducing inflammation	(104)
melasma	√				epidermal keratinocytes	stimulating keratinocyte differentiation and influencing redox leveling	effects on melanosome transfer and melanogenesis	(105)
Rosacea	√				epidermal keratinocytes	influencing Cathelicidin and VEGF	induction of inflammation	(109)
Rosacea	√				epidermal keratinocytes and neurons	influencing TRPV1 on vasoregulation and nociception	neurogenic inflammation	(110)
Acne	√				sebaceous glands	/	sebum secretion and induction of immunity;	(111)
Allergic contact dermatitis	√		√		epidermal keratinocytes and myeloid cells	promoting the development of T cell	inducing immunity	(113)
Dermatomyositis	√		√		peripheral blood mononuclear cells (PBMCs) and muscle tissues	altering the cytoskeleton of dermal microvascular endothelial cells	inducing immunity, inflammation	(115)
Skin photoaging	√				epidermal keratinocytes	promoting inflammatory responses through Akt-mediated phosphorylation of NF-κB along with FoxO6 (Ser184), and suppressing the antioxidant enzyme MnSOD,	inducing inflammation and ROS	(117)
Other chronic pruritic conditions(e.g., cowhage, spicules-induced, dermatophyte-associated and scabies itch)	√				epidermal keratinocytes and neurons	releasing ion channels (e.g.TRPV3) and promotion of Th2 cytokines	induction of neurogenic pruritus and inflammation	(120)
	√		√		epidermal keratinocytes and mast cells	amplification of neuro-epidermal communication and inflamatory responses	induction of immunity inflammation and itch	(121, 122)

### 4.1 Atopic dermatitis

Atopic dermatitis, also known as atopic eczema, is the most common inflammatory skin disorder, characterized by genetic barrier defects, allergic inflammation and intractable pruritus (77). Recent consensus illustrates that it may be a systemic disease involving multiple allergic and respiratory comorbidities (78). The mechanisms are not well understood, but they are believed to influence neuro-immune and neuro-epidermal communications in the local microenvironment (79). Several genes related to epidermal barrier homeostasis, including SPINK5, and filaggrin, have been identified as abnormal in AD, resulting in elevated skin pH and increased penetration of allergens through the defective skin barrier. SPINK5 encodes lympho-epithelial Kazal-type-related inhibitor (LEKTI) (80), a major inhibitor of KLKs. Studies suggest that single nucleotide polymorphisms E420K and D386N of SPINK5 reduce LEKTI function, thus up-regulating KLKs expression in AD patients. Accordingly, endogenous serine proteases of PAR2 (e.g. KLK5 and KLK14) are active, facilitating easier allergen penetration through the skin barrier in AD (81). Many reports have revealed elevated expression and activation of PAR2 in the lesional skin of AD patients (82).Upon stimulation, PAR2 in epidermal keratinocytes and peripheral nerves leads to releasing Th2 cytokines, intensifying inflammation by attracting immune cells, and initiating neurogenic inflammation associated with itching sensation (55, 83, 84). Owing to active KLKs in AD, PAR2 also indirectly correlates with the regulation of antimicrobial peptides, which are key for innate immunity (83). Moreover, a transgenic mouse model overexpressing epidermal PAR2 presents AD-like appearance, with enhanced PAR2 in nerve fibers contributing to itching behavior due to direct neuro-epidermal communication (85). Another study indicated that elevated PAR2 expression on nerve fibers prompted itching following the application of PAR2 agonists (86). Briefly, current data focus primarily on keratinocytes and slightly on neurons, without exploration of the role of PAR2 on immune and inflammatory cells. In a study by Smith, after treatment with HDM, model mice with epidermal overexpression of PAR2, particularly cell-specific, exhibited typical AD symptoms and significant infiltration of mast cells and eosinophils, though there was no deeper investigation into PAR2’s role in these cells (87). Thus, we infer that PAR2 is implicated in inflammation, pruritus, and barrier regulation in AD, but also affects relevant comorbidities caused by overactive mast cells.

These findings illustrate that PAR2 could be a promising therapeutic target in AD. Studies on PAR2 antagonists, including ENMD-1198 (88) and NPS-1577 (89), have demonstrated varying degrees of alleviation in AD symptoms. In 2019, Barr et al. demonstrated that PZ-235 could be a promising option for AD by targeting neuro-immune interactions *in vivo*, thereby reducing scratching behavior, attenuating the production of inflammatory-immune factors, and decreasing lesion severity (90). Furthermore, latest data reveal that topical doxycycline monohydrate hydrogel, which downregulates PAR2 activity, exhibits significant clinical efficacy in AD patients (91).

### 4.2 Psoriasis

Psoriasis is a prevalent chronic inflammatory dermatological condition, characterized by a multifactorial etiology involving both immune dysregulation and genetic predispositions. In genetically susceptible individuals, various external and internal stimuli activate the immune system, triggering a series of cellular responses that include the participation of plasmacytoid dendritic cells, macrophages, mast cells and T cells. The immune activation results in the hyperproliferation and aberrant differentiation of keratinocytes, as well as severe pruritus (92). Current research has predominantly focused on the role of PAR2 in plaque psoriasis, revealing differential expression levels of PAR2 across various cell types within psoriatic lesions. In previous studies, patients with psoriasis vulgaris have exhibited lower levels of PAR2 in keratinocytes. This reduction may be attributed to a process of PAR2 internalization, where excessive stimulation promotes PAR2-mediated IL-8 production, leading to an accumulation of inflammatory cells in the epidermis without sufficient PAR2 replenishment (93). In the context of mast cells, Carvalho et al. demonstrated a significant increase in PAR2 levels in the lesional skin of psoriasis patients compared to healthy skin. This could result from the persistent activation of mast cells, which is a characteristic feature of psoriasis. Moreover, interaction of PAR2-activating peptides with mast cells results in elevated secretion of IL-8 rather than histamine release (94). Notably, Nattkemper et al. recently discovered that epidermal expression of PAR2 was significantly increased in scalp psoriasis accompanied by severe itch. The difference may be due to the distinct distribution of PAR2 across different body areas, as it is present on sensory nerve endings, epidermal keratinocytes, and the inner root sheath (IRS) in scalp hair follicles (95). Furthermore, a study measuring PAR2 levels over time in psoriasis patients treated with a combination of ultraviolet rays and methotrexate reported a significant decrease in PAR2 levels. This decrease may result from alterations in antigen-presenting cells, intracellular signaling pathways, and anti-inflammatory processes induced by this combined therapy. However, the study did not clarify which specific cell types had varying PAR2 levels (96). These pieces of evidence indicate that PAR2 in psoriasis exhibits diverse effects depending on its location in different cell types-lower expression in keratinocytes versus higher expression in mast cells and enhanced epidermal expression of PAR2 in scalp-indicating a closely correlated pathophysiology involving multiple cell variants in the cutaneous microenvironment. In this context, immune and inflammatory cells closely interact with basal keratinocytes or adjacent blood vessels in the dermis. Consequently, PAR2 antagonists may present a potential therapeutic strategy for managing inflammation and itch associated with psoriasis.

### 4.3 Vitiligo

Vitiligo is an autoimmune skin disorder characterized by the loss of functional melanocytes, resulting in white patches on the skin and mucous membranes, as well as white hair (97). It is increasingly recognized as a systemic disease with various comorbidities, such as AD, alopecia areata, and systemic lupus erythematosus (98). Currently, the essential pathogenesis involves persistent oxidative stress resulting from dysfunction in the nuclear factor erythroid 2-related factor 2 pathway, along with autoimmunity stemming from hyperactive innate and adaptive immune responses. Consequently, treatments have primarily targeted antioxidants and immunosuppressants (99). In recent years, PAR2 has been identified as a key player in the pathogenesis of vitiligo, despite not being present in melanocytes. In 2009, Moretti and colleagues (100) first discovered that PAR2 levels were significantly reduced in the lesions of white patches compared to non-lesional skin in vitiligo. Interestingly, this phenomenon was not observed in other non-vitiligo depigmentation conditions, such as pityriasis versicolor and lichen simplex chronicus. This implies that PAR2 downregulation is specific to vitiligo-related depigmentation. The reduced PAR2 may impair the function of keratinocytes in white patches, including the inhibition of melanosome transfer to neighboring cells. Kim et al. later illustrated that PAR2 can enhance Nrf2-mediated antioxidant responses, protecting the skin from excessive oxidative damage and thus maintaining pigmentation through interactions between keratinocytes and melanocytes (101). Their study may explain the lower levels of PAR2 in vitiligo. Additionally, Tang found that phototherapy such as narrow band, may regulate pigmentation in vitiligo by affecting PAR2 on keratinocytes, influencing melanosome uptake and malondialdehyde level (102). Collectively, the decrease in PAR2 plays a critical role in the pathogenesis of vitiligo. Further research is necessary to understand whether PAR2 influences immunity and whether other cells, such as mast cells and T cells, undergo similar PAR2 changes in vitiligo. Understanding the exact mechanism of PAR2 may enhance our knowledge of the crosstalk between melanocytes and their surrounding cells, inform the potential for comorbidities, and aid in the development of effective therapeutic strategies for vitiligo.

### 4.4 Melasma

Melasma is a common chronic acquired hyperpigmentation disorder that usually affects photoexposed areas in predisposed individuals, with ultraviolet (UV) radiation being the primary risk factor. Although the exact pathogenesis remains unclear, it is acknowledged that melasma originates from alterations in several cell types, including melanocytes, keratinocytes and mast cells. These changes lead to the production and transfer of mature melanosomes throughout the epidermis (103). PAR2 has been increasingly recognized as a significant contributor to the pathogenesis of melasma, especially in melanosome transfer and melanogenesis through a specific paracrine mechanism. For instance, Lee et al. discovered that the PAR2 expression was increased in melasma patients and positively correlated with clinical telangiectatic erythema. Upregulation of PAR2 by VEGF stimulation was clearly evident, suggesting that abnormal PAR2 activity may facilitate inflammatory erythema (104). A recent study by Kim et al. indicated that PAR2 might be involved in a series of reactions involving the NRF2 pathway, which subsequently inhibits primary cilia formation and the Hedgehog signaling pathway, while also stimulating keratinocyte differentiation. These processes ultimately lead to increased melanin synthesis and excessive transfer of melanosomes to keratinocytes in melasma (105). Moreover, UV radiation was found to upregulate epidermal PAR2 expression and proteolysis, with notable variations among different skin phototypes (106). Given that mast cells degranulate under UV radiation, it is inferred that PAR2 present in mast cells may also influence the associated pathogenesis of melasma. Therefore, promising PAR2 antagonists may offer a novel therapeutic approach for the treatment of melasma. Further research is needed to fully elucidate the role of PAR2 in melasma.

### 4.5 Others

Rosacea is a chronic inflammatory dermatosis characterized by facial flushing, telangiectasia, inflammatory papules and pustules, primarily affecting the central face. Neurovascular and neuroimmune dysregulation are significant contributors to the mechanisms underlying rosacea (107). External stimuli such as heat or alcohol can exacerbate the condition due to heightened skin sensitivity. Among the factors involved, the cathelicidin LL-37 (an antimicrobial peptide) activation pathway is the best understood and most classical pathway in rosacea pathogenesis (108). In 2014, a positive correlation between PAR2 and cathelicidin was observed in rosacea patients. Additionally, treatment with PAR2-activating peptides *in vitro* led to increased levels of cathelicidin and VEGF (109). Moreover, TRPV1, found on neurons and keratinocytes, was activated via upregulated PAR2 in rosacea (110). PAR2 also appears to influence the development of acne. A study by Lee et al. found greater PAR2 expression in sebaceous glands, rather than the epidermis, in inflammatory acne lesions (111). Allergic contact dermatitis (ACD), a type IV hypersensitivity reaction, often requires treatment to reduce inflammation induced by re-exposure to allergens (112). Remarkably, a latest study revealed that myeloid cells expressed increased PAR2 in human ACD, promoting the development of T cell-mediated inflammation (113). Dermatomyositis, a rare autoimmune disease characterized by skin rash and muscle weakness, was found to involve increased levels of Cathepsin G in peripheral blood mononuclear cells and muscle tissues. This increase correlated with disease severity and was found to induce PAR2 secretion, suggesting an indirect role for PAR2 (14–115). Skin photoaging arises from long-term exposure to UV irradiation, leading to ROS production and inflammatory responses (116). A 2021 report illustrated that active PAR2 in keratinocytes promoted inflammatory responses through Akt-mediated phosphorylation of NF-κB and FoxO6, while also suppressing the antioxidant enzyme MnSOD, thereby progressively increasing ROS levels (117). Drawing from the research by Piran et al (56–58), We speculate that active PAR2 here was related to both T-lymphocyte-mediated immune inflammation and PAR2 activation within the affected keratinocytes themselves. PAR2 could eventually aggravate inflammation, suggesting that the two processes above may have a synergistic effect in skin photoaging, where tissue regeneration is not involved. PAR2 is also implicated in various chronic pruritic conditions, particularly in histamine-independent pruritus caused by cowhage spicules, dermatophytes, and scabies (118). The pathogenesis of pruritus involves a complex network of interactions among keratinocytes, sensory neurons, mast cells and transiently infiltrating immune cells (119). Emerging reports suggest that keratinocytes act as the initial sensor for itch signaling, and that interaction with various cells excessively exacerbates inflammation and itching. Activation of epidermal PAR2 triggers intracellular PLC-Ca^2+^ signaling, leading to TSLP-relevant scratching behavior. TSLP then promotes the generation of type 2 cytokines and stimulates PAR2, TRPV1, and TRPA1 in sensory neurons, exacerbating itch responses. Additionally, PAR2 activation in dorsal root ganglia enhances the function of epidermal TRPV3, perpetuating the itch-scratch cycle (120). In a study by Park et al (121), the PAR2-TRPV3-TSLP pathway was identified as critical in the pruritus experienced by burn scar patients. Another study by Kristen et al. found that PAR2 expression was significantly increased in the epidermis and mast cells near the dermal-epidermal junction in scabies-infested tissues, explaining why conventional antihistamines are often ineffective against scabies itch (122). Collectively, these findings highlight the potential of PAR2 agonists and antagonists for developing new therapeutic strategies that could not only address the limitations of classical antipruritics but also circumvent the side effects associated with topical corticosteroids.

## 5 Conclusion

In summary, our comprehension of the role of PAR2 in cutaneous immune and inflammatory processes is advancing swiftly, uncovering novel insights and potential therapeutic targets. This review underscores several critical aspects: (a). The proteolytic activation of PAR2 at different sites initiates intricate signaling cascades, emphasizing the importance of biased activation for a deeper understanding of its roles in specific diseases. (b). PAR2, present in various cells within the cutaneous microenvironment, acts as a multifaceted target for interactions among sensory neurons, resident skin cells, and transiently infiltrating cells. Accordingly, potent PAR2 agonists and antagonists hold promise for addressing the complexities of inflammatory and autoimmune dermatological diseases. (c). PAR2 plays a crucial role in elucidating neuro-immune and immune-inflammatory interactions in these conditions, thereby offering valuable insights into the mechanisms underlying their diverse cutaneous and extracutaneous comorbidities.
